# Expanding Puck and Schürmann Inter Fiber Fracture Criterion for Fiber Reinforced Thermoplastic 3D-Printed Composite Materials

**DOI:** 10.3390/ma13071653

**Published:** 2020-04-02

**Authors:** Thiago Assis Dutra, Rafael Thiago Luiz Ferreira, Hugo Borelli Resende, Brina Jane Blinzler, Ragnar Larsson

**Affiliations:** 1 GPMA-Research Group on Additive Manufacturing, DCTA ITA IEM, ITA-Aeronautics Institute of Technology, São José dos Campos, São Paulo 12228-900, Brazil; 2 Division of Material and Computational Mechanics, Department of Industrial and Materials Science, Chalmers University of Technology, SE-412 96 Gothenburg, Sweden; 3 LEL-Lightweight Structures Laboratory, IPT-Institute for Technological Research, São José dos Campos, São Paulo 12247-016, Brazil

**Keywords:** 3D-printed composite materials, additive manufacturing, failure of composites, puck and schürmann failure criterion

## Abstract

The present work expands the application of Puck and Schürmann Inter-Fiber Fracture criterion to fiber reinforced thermoplastic 3D-printed composite materials. The effect of the ratio between the transverse compressive strength and the in-plane shear strength is discussed and a new transition point between the fracture conditions under compressive loading is proposed. The recommended values of the inclination parameters, as well as their effects on the proposed method, are also discussed. Failure envelopes are presented for different 3D-printed materials and also for traditional composite materials. The failure envelopes obtained here are compared to those provided by the original Puck and Schürmann criterion and to those provided by Gu and Chen. The differences between them are analyzed with the support of geometrical techniques and also statistical tools. It is demonstrated that the Expanded Puck and Schürmann is capable of providing more suitable failure envelopes for fiber reinforced thermoplastic 3D-printed composite materials in addition to traditional semi-brittle, brittle and intrinsically brittle composite materials.

## 1. Introduction

Traditional composite materials, in particular continuous fiber reinforced plastics, are able to provide excellent mechanical properties in addition to a large potential for optimization [1,2,3,4]. A traditional composite manufacturing challenge, however, is inclusion of the reinforcing fibers into the polymer matrix with good consolidation, control of fiber orientation and low cost [5]. In general, manufacturing of traditional composite materials involves process-stages where the material is laid-up over a mold prior to cure or consolidation, frequently under high temperature and pressure, which increases the manufacturing costs. In this context, several modern alternative composite manufacturing processes have been investigated recently, among which Additive Manufacturing (AM) of composite materials, although still under development, has demonstrated potential to produce functional parts in a more cost effective and faster manner [6].

**Figure 1:**
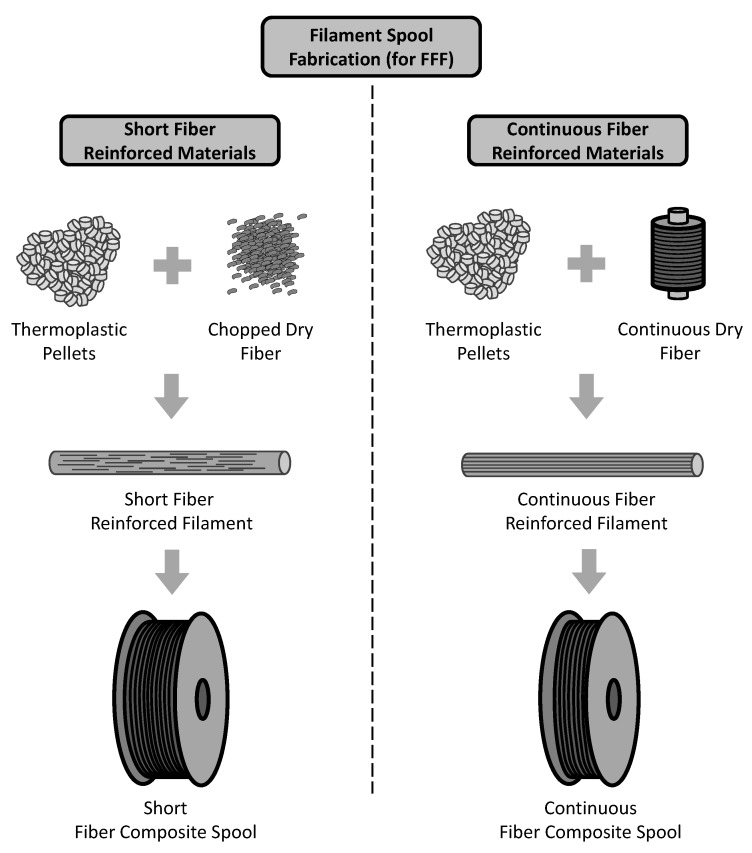
Schematic view of differences on the fabrication of short fiber reinforced 3D-printing filaments compared to continuous fiber reinforced 3D-printing filaments.

Among the processes contemplated in AM [7], the material extrusion based technology Fused Filament Fabrication (FFF) has been playing an important role. This can be attributed to several aspects among which it can be highlighted the high offer of open source machines with affordable costs, the availability of open access information on software and hardware and possibility of production of parts with some mechanical responsibility. Consequently, its application for final parts has been studied for different sectors over the last years [8,9,10,11]. Additional details about the traditional FFF process are presented by Guo and Leu in [6]. In regards to the feedstock material, the FFF process, which is also referred by the general term 3D-printing, is currently able to produce thermoplastic fiber reinforced parts in addition to the initial unreinforced ones. The most common fiber reinforced thermoplastic filaments contain short reinforcing fibers, although continuous reinforcing fibers have also been successfully introduced into this technology [12,13]. Figure 1 depicts the basic differences in the fabrication of short fiber reinforced filaments compared to continuous fiber reinforced filaments. It must be noted that different impregnation processes can be applied in order to blend the thermoplastic matrix with the reinforcing fibers. Although the length of the chopped dry fibers may vary from one supplier to another, normally they are very small in order to prevent clogging issues during the extrusion process, e.g., Ning et al. reported in [14] that chopped dry fibers had length of 100μm and 150μm, and Ferreira et al. [15] reported chopped dry fibers of length 60μm. Another limitation of using short reinforcing fibers is the fiber volume content as Wang et al. reported in [13]. Thus, the introduction of continuous reinforcing fibers has been adopted as an option to produce stiffer and stronger parts.

**Figure 2:**
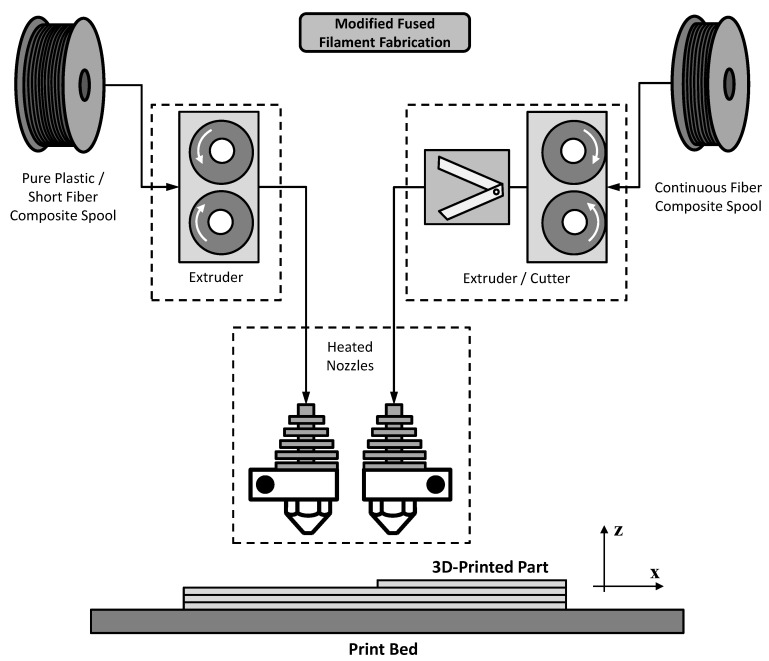
Schematic view of modified FFF process which is able to use unreinforced filaments or chopped fiber reinforced filaments (typical extrusion system) and continuous fiber reinforced filaments (specific extrusion and cutting system).

The introduction of high performance materials in 3D-printing initially required significant investigation into their mechanical behavior. It is well-established in the literature that their resulting mechanical properties also depend on the process parameters in addition to the individual constituent properties [16,17,18,19,20,21,22]. Many researchers have been working on determining the mechanical properties of short fibers reinforced 3D-printed materials [14,15,23,24,25,26]. However, very recent research has been conducted on the investigation of continuous fiber reinforced 3D-printed materials either adapting conventional printers [27,28,29,30,31] or using commercial solutions [5,32,33,34,35,36,37,38,39,40]. Figure 2 illustrates a schematic view of the FFF process modified to use continuous fiber reinforced filaments [41]. Similar to the traditional FFF process, the modified FFF process also requires a 3D computer aided design (CAD) model which is initially sliced into successive layers where the thickness is defined according to the final thickness of deposited reinforced layers. The part is also built bottom up, one layer at a time. In opposition to adapting conventional printers to work with continuous reinforcing fibers, in commercial solutions there may exist some constraints to configure printing parameters such as infill speed, nozzle extrusion temperature and infill type [41]. In contrast to short fiber reinforced filaments, which can be used with common extrusion based 3D-printers, the continuous fiber reinforced filaments require the incorporation of additional features to the 3D-printers such as specific extruders and cutting systems among others. The pre-tension applied to the fiber spool is also a concern since the filaments are stiffer than regular ones, and consequently have a higher potential to become unspooled.

It can be realized from the material constitutive relation point of view [5,32,33,34,35,36,37,38,39,40] that continuous fiber reinforced 3D-printed materials exhibit similar characteristics to traditional orthotropic unidirectional composites, i.e., the resulting mechanical properties along to the fiber direction are substantially higher than the mechanical properties transverse to fiber direction. Even unreinforced and short fiber reinforced 3D-printed materials exhibit an orthogonal material behavior as can be seen in [15,16,42,43].

In spite of the recent investigations, a gap still remains regarding detailed studies about the failure of 3D-printed materials, as well as the recommended failure criteria. For instance, Uddin et al. [44] evaluated the failure mechanisms of 3D-printed unreinforced ABS. However, an approach considering traditional failure criteria could be seen only in very recent work [45] where it was applied the well-known Tsai-Hill anisotropic yield criterion in the prediction of the ultimate tensile strength of 3D-printed PLA. In the context of continuous fiber reinforced 3D-printed materials, the progress of these investigations are slightly behind when compared to the unreinforced ones, e.g., recent work only investigated some aspects of fracture mechanisms for continuous fiber reinforced 3D-printed materials as can be seen in [37,39,46]. Albeit there is still a certain lack of studies about the recommended failure criteria for 3D-printed materials, the authors agree that in order to understand the failure criteria in fiber reinforced thermoplastic 3D-printed composite materials, it is firstly necessary to know the fracture mechanisms.

For traditional composite materials, classical lamina failure criteria are based on strengths under fundamental loads (tension and compression in longitudinal and transverse directions and in-plane shear) and may be classified as non-interactive, interactive and partially interactive [47]. Among the classical failure criteria, it can be cited the Maximum Stress, Maximum Strain, Azzi-Tsai, Tsai-Wu and Hashin. More detailed information about these failure criteria, in addition to other criteria, can be found in [47,48,49,50,51]. Whilst providing failure envelopes for composite laminae, these previous failure criteria lack of phenomenological background which might lead to non-expected solutions for cases outside of their designed purpose. Viewing to circumvent this issue and also to provide a better definition of what constitutes the failure of a composite, a World Wide Failure Exercise (WWFE) was proposed by Hinton and Soden [52]. Many researchers were invited to this failure exercise. According to Soden et al. [53] one of the leading theories was presented by Puck and Schürmann in [54,55], which was complemented afterwards by Puck et al. in [56]. This theory assumes two distinguished types of fracture, i.e., Fiber Fracture (FF) and Inter-Fiber Fracture (IFF).

The Puck and Schürmann Inter-Fiber Fracture (IFF) criterion is based on a modified Mohr-Coulomb criterion for brittle materials with the advantage of carrying a phenomenological basis. In particular, this theory allows the prediction of the fracture plane and consequently assesses the failure under both transverse tension and compression. For this purpose, the Puck and Schürmann failure criterion [54,55] requires, in addition to the strengths under fundamental loads, the computation of other parameters [57]. These parameters are related to the fracture resistances of the action plane ($R_{⊥}^{A}$, $R_{⊥⊥}^{A}$ and $R_{⊥\|}^{A}$) and to the inclination at $\sigma_{n}=0$ for some sections of the master fracture body ($p_{⊥\|}^{c}$, $p_{⊥\|}^{t}$, $p_{⊥⊥}^{c}$ and $p_{⊥⊥}^{t}$). Detailed information about these parameters are presented in [54,55,56,57].

As a consequence of its phenomenological basis, besides the excellent agreement with experimental data, the Puck and Schürmann failure criterion [54,55] is largely used nowadays in the composite field either in its original form or as a basis for extended theories as can be seen in the literature [58,59,60,61,62,63,64,65,66,67]. For instance, the fracture plane concept proposed by Puck and Schürmann [54,55] was taken into account to develop the LaRC03 criterion in [68] where a set of six nonempirical criteria for predicting failure of unidirectional fiber reinforced plastic laminates is described. Although, the LaRC03 criterion does not require several parameters that are not physical, additional unidirectional properties are required, e.g., $G_{Ic}$ and $G_{IIc}$ which could not be seen in the available literature for 3D-printed composite materials. In terms of use and application, it can be highlighted that the Puck and Schürmann failure criterion [54,55] is surprisingly straightforward and became an important tool for use in the quotidian of engineering design of laminated composite parts and components. More specifically, its application is very practical for the industry, not only in preliminary design, but also in critical and final design reviews since it is able to provide results for the stress level resulting in crack initiation and fracture as well as indicating the direction of cracks, mostly requiring simple analytical calculations after stress field computations. Consequently, it contributes to a more accurate design of laminated composite parts and saves computational time and cost.

Although there are many qualities and advantages, a limitation of the Puck and Schürmann failure criterion is that it was initially developed for intrinsically brittle materials. It means that its application in predicting the failure envelopes for some types of fiber reinforced thermoplastic materials may require adjustments on its specific parameters. Based on the results of the present work, in addition to the results found in recent literature, fiber reinforced thermoplastic 3D-printed composite materials are certainly included in these types of materials. For instance, according to Verdejo de Toro et al. [69] the FFF process is responsible to promote changes in the cristallinity of thermoplastic matrix. Moreover, these changes showed to have more effect over the mechanical properties measured in tension rather than in compression. In other recent work, Pascual-González et al. [70] characterized the composition and calorimetric properties of continuous and short fiber reinforced raw filaments, in addition to unreinforced raw filaments, before printing. It was verified that the continuous fiber reinforced composite filaments can present an amorphous nature before printing in opposition to semi-crystalline nature found in thermoplastic matrix commonly used in FFF process. Therefore, the changes in the cristallinity promoted by the FFF process can affect the ratio $Y_{C}/Y_{T}$. As consequence, these ratios can be significantly different for 3D-printed composite materials, when compared to traditional composite materials, requiring the adjustments previously mentioned.

Very recently, Gu and Chen [71] presented an extension of Puck and Schürmann failure criterion which was expected to be applicable for unidirectional composites with different $Y_{C}/Y_{T}$ ratios than those originally presented in [54,55,56]. Based on this, Gu and Chen extension [71] should also be able to predict the failure envelopes of fiber reinforced thermoplastic 3D-printed composite materials. However, Gu and Chen [71] only focused on the inclination parameters that are related to the transverse shear, $\tau_{23}$, which was estimated, since it is difficult to be experimentally measured. Furthermore, nothing was mentioned about the ratio $Y_{C}/S_{12}$ which can be very different for fiber reinforced thermoplastic 3D-printed composite materials when compared to traditional composites, as can be seen in the available literature [5,15,35,40]. Thus, it may be observed that the proper application of Puck and Schürmann failure criterion to 3D-printed continuous fiber reinforced thermoplastics still remains a gap in the literature.

### Objective and Contribution

The present work aims to expand the application of Puck and Schürmann Inter-Fiber Fracture criterion to 3D-printed continuous fiber reinforced composite materials providing a natural step forward in the analysis of these materials which is expected for both the scientific community and industry. As earlier noted, high performance 3D-printed composite materials, in particular the continuous fiber reinforced thermoplastics, are playing a remarkable role in the AM context and their importance is growing daily, due to their potential. It follows that, understanding and predicting their mechanical behavior is essential for the accurate design of components. However, it can be seen in literature that there still exists investigation gaps, mostly in failure prediction. In view of these aspects, the present work contributes providing the baseline necessary to predict the failure of fiber reinforced thermoplastic 3D-printed composite materials based on a well-known failure criterion, i.e., Puck and Schürmann. To this end, the present work describes a methodology to determine the Inter-Fiber Fracture failure conditions taking into account the ratios between the transverse compressive and tensile strength as well as the transverse compressive and in-plane shear strength. For the sake of clarification, resulting failure envelopes are presented for different 3D-printed materials as well as for traditional materials. The effect of the inclination parameters on the proposed method is also discussed. The obtained failure envelopes are then compared to the envelopes provided by the Puck and Schürmann failure criterion [54,55] and also to those provided by Gu and Chen [71].

There are several motivations of using Puck and Schürmann failure criterion in order to predict the failure envelopes of fiber reinforced thermoplastic 3D-printed composite materials. Here the most relevant are discussed. Firstly, it can be seen that the Puck and Schürmann failure criterion is able to distinguish the different mode of failures in Inter-Fiber Fracture and for intrinsically brittle thermoset based composite materials it presents formidable agreement with experimental data. Secondly, as previously mentioned, in function of its phenomenological basis it has been widely used in the composite materials field even as a baseline for further adaptations or serving as a benchmark to novel propositions. From the statement that fiber reinforced thermoplastic 3D-printed composite materials are orthotropic materials, specially for continuous fiber reinforced cases, the Puck and Schürmann failure criterion has the ability to provide good results in predicting the failure envelopes for these materials. Lastly, the authors believe that this type of approach is of high relevance for fiber reinforced thermoplastic 3D-printed composite materials since it provides tools for the next step in the analysis of 3D-printed parts, i.e., rigorous failure analysis, which could not be seen in the available literature. Consequently, it would enhance their possibilities of application in different other sectors.

In regards to the paper structure, an overview about the Puck and Schürmann Inter-Fiber Fracture criterion is presented in the next section. This overview is complemented by its application to a fiber reinforced thermoplastic 3D-printed composite materials and more details about its limitations are provided. In Section 3 the Expanded Puck and Schürmann Inter-Fiber Fracture criterion is presented. In this section the adopted assumptions as well as the failure conditions and their respective equations are described. Examples of failure envelopes computed for 3D-printed materials and also for traditional composite materials are presented in Section 4 and Section 5 respectively. The main aspects of the computed failures envelopes are discussed in these sections. In Section 6, the differences between the computed failure envelopes are analyzed with the support of geometrical techniques and also statistical tools. The concluding remarks about the present work are presented in Section 7.

## 2. Puck and Schürmann Inter-Fiber Fracture Criterion

**Figure 3:**
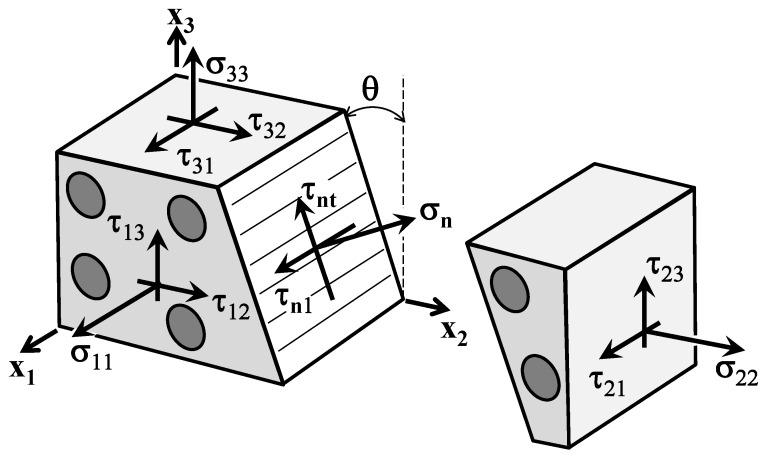
Stress state associated with fiber reinforced composites where $\sigma_{n}$, $\tau_{nt}$ and $\tau_{n1}$ are the stresses on the action plane and θ is the rotation of 2-face about the 1-axis (Adapted from [57]).

The Puck and Schürmann Inter-Fiber Fracture criterion is based on the assumption that the stresses acting on a fracture plane will induce fracture. Thus, the theory is formulated in function of the stresses $\sigma_{n}\left(\theta \right)$, $\tau_{nt}\left(\theta \right)$ and $\tau_{n1}\left(\theta \right)$ [54,55,56,57] as can be seen in Figure 3. The stress components in Figure 3 are related to the fiber orientation, where the 1-index is pointing to the fiber direction. The fracture angle θ is a rotation of the stress action plane, i.e., the rotation of 2-face about the 1-axis. In order to visualize the stresses acting on the fracture plane, a 3D stress state is shown in Figure 3. However, in the next sections, this formulation is derived to a plane stress case since the stresses in thickness direction of the lamina (3-axis) will be neglected.

Taking into account Figure 3, the stresses $\sigma_{n}\left(\theta \right)$, $\tau_{nt}\left(\theta \right)$ and $\tau_{n1}\left(\theta \right)$ can be defined as:

(1a) $$\begin{array}{cc} \; & \sigma_{n}\left(\theta \right)=\sigma_{22}\cos^{2}\left(\theta \right)+\sigma_{33}\sin^{2}\left(\theta \right)+2\tau_{23}\sin\left(\theta \right)\cos\left(\theta \right), \end{array}$$

(1b) $$\begin{array}{cc} \; & \tau_{nt}\left(\theta \right)=-\sigma_{22}\sin\left(\theta \right)\cos\left(\theta \right)+\sigma_{33}\sin\left(\theta \right)\cos\left(\theta \right)+\tau_{23}\left[\cos^{2}\left(\theta \right)-\sin^{2}\left(\theta \right)\right], \end{array}$$

(1c) $$\begin{array}{cc} \; & \tau_{n1}\left(\theta \right)=\tau_{13}\sin\left(\theta \right)+\tau_{12}\cos\left(\theta \right). \end{array}$$

The stresses $\sigma_{n}\left(\theta \right)$, $\tau_{nt}\left(\theta \right)$ and $\tau_{n1}\left(\theta \right)$, as well as the risk of fracture, depend on the action plane which is oriented according to the angle θ (see Figure 3). Thus, the fracture occurs in the plane with the highest stress exposure $f_{E}\left(\theta_{fp}\right)=\max_{\theta }f_{E}\left(\theta \right)$. For a given stress-state, the action plane with the highest stress exposure $f_{E}\left(\theta_{fp}\right)$ is computed from all rotated planes within the interval [−90°,90°] where $\theta_{fp}=\arg\max_{\theta }f_{E}\left(\theta \right)$, i.e., $\theta_{fp}=\left\{\theta_{fp}|\forall \theta :f_{E}\left(\theta \right)\leq f_{E}\left(\theta_{fp}\right)\right\}$.

The fracture condition is then satisfied when failure initiation occurs, i.e., when the stress exposure $f_{E}\left(\theta \right)\geq 1$. The fracture exposure can be written as [57]:

(2a) $$\begin{array}{cc} \; & f_{E}\left(\theta \right)=\sqrt{{\left[\left(\frac{1}{R_{⊥}^{A}}-\frac{p_{⊥\psi }^{t}}{R_{⊥\psi }^{A}}\right)\sigma_{n}\left(\theta \right)\right]}^{2}+{\left[\frac{\tau_{nt}\left(\theta \right)}{R_{⊥⊥}^{A}}\right]}^{2}+{\left[\frac{\tau_{n1}\left(\theta \right)}{R_{⊥\|}^{A}}\right]}^{2}}+\frac{p_{⊥\psi }^{t}}{R_{⊥\psi }^{A}}\sigma_{n}\left(\theta \right),\mathrm{for}\sigma_{n}\left(\theta \right)\geq 0, \end{array}$$

(2b) $$\begin{array}{cc} \; & f_{E}\left(\theta \right)=\sqrt{{\left[\frac{p_{⊥\psi }^{c}}{R_{⊥\psi }^{A}}\sigma_{n}\left(\theta \right)\right]}^{2}+{\left[\frac{\tau_{nt}\left(\theta \right)}{R_{⊥⊥}^{A}}\right]}^{2}+{\left[\frac{\tau_{n1}\left(\theta \right)}{R_{⊥\|}^{A}}\right]}^{2}}+\frac{p_{⊥\psi }^{c}}{R_{⊥\psi }^{A}}\sigma_{n}\left(\theta \right),\mathrm{for}\sigma_{n}\left(\theta \right)<0, \end{array}$$

with

(3a) $$\begin{array}{cc} \; & \frac{p_{⊥\psi }^{t,c}}{R_{⊥\psi }^{A}}=\frac{p_{⊥⊥}^{t,c}}{R_{⊥⊥}^{A}}\cos^{2}\psi +\frac{p_{⊥\|}^{t,c}}{R_{⊥\|}^{A}}\sin^{2}\psi , \end{array}$$

(3b) $$\begin{array}{cc} \; & \cos^{2}\psi =\frac{\tau_{nt}^{2}}{\tau_{nt}^{2}+\tau_{n1}^{2}}, \end{array}$$

(3c) $$\begin{array}{cc} \; & \sin^{2}\psi =\frac{\tau_{n1}^{2}}{\tau_{nt}^{2}+\tau_{n1}^{2}}, \end{array}$$

(3d) $$\begin{array}{cc} \; & R_{⊥⊥}^{A}=\frac{R_{⊥}^{c}}{2\left(1+p_{⊥⊥}^{c}\right)}, \end{array}$$

(3e) $$\begin{array}{cc} \; & R_{⊥}^{c}=Y_{C}, \end{array}$$

(3f) $$\begin{array}{cc} \; & R_{⊥}^{A}=R_{⊥}^{t}=Y_{T}, \end{array}$$

(3g) $$\begin{array}{cc} \; & R_{⊥\|}^{A}=S_{12}, \end{array}$$

where $Y_{T}$ and $Y_{C}$ are respectively the tensile and compressive strength of a unidirectional layer transverse to the fiber direction and $S_{12}$ is the in-plane shear strength of a unidirectional layer.

Besides the basic strengths, the parameters $p_{⊥⊥}^{t,c}$ and $p_{⊥\|}^{t,c}$ are introduced in Equation (3a), Equation (3d). These parameters, also called inclination parameters, are the slope of the $\left(\sigma_{n},\tau_{nt}\right)$ and $\left(\sigma_{n},\tau_{n1}\right)$ fracture envelopes respectively. Typical values for the inclination parameters $p_{⊥\|}^{c}$, $p_{⊥\|}^{t}$, $p_{⊥⊥}^{c}$ and $p_{⊥⊥}^{t}$ were recommended by Puck et al. in [56]. These values of inclination parameters were obtained for intrinsically brittle composite materials and are here listed in Table 1. According to Knops [57], the recommended values in Table 1 do not cause problems, e.g., discontinuous curves or non-capture of the experimental points, for most reinforced fiber composite materials containing a thermoset matrix. For the remaining exceptional cases a relation between the inclination and the resistances of the stress action plane shall be used. However, Knops [57] remarked that values not lower than $p_{⊥⊥}^{c}=0.2$ should be used since unrealistic transverse compression fracture angles would be computed. Establishing this lower limit for $p_{⊥⊥}^{c}$, means that for materials whose ratio $Y_{C}/Y_{T}$ are significantly small, the fracture angle might be $\theta_{fp}\neq 45{}^{\circ}$ for a pure $\tau_{23}$ stress.

### 2.1. Preliminary Inspection for Intrinsically Brittle Materials

According to Gu and Chen [71], the unidirectional fiber reinforced composites can be categorized into semi-brittle, brittle and intrinsically brittle materials. Table 2 summarizes the classification which accounts to the ratio between the transverse compressive strength and the transverse tensile strength.

**Figure 4:**
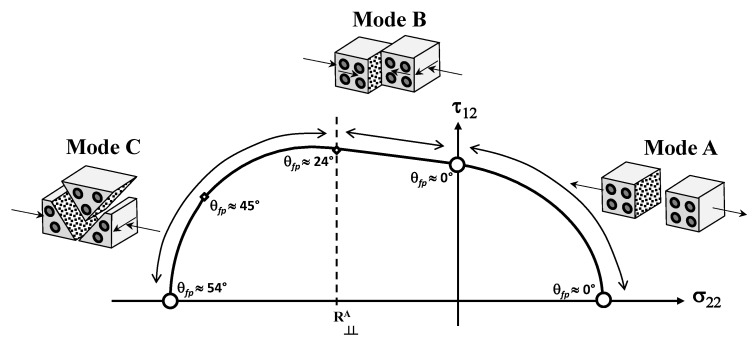
Fracture curve on the stress space $\sigma_{22}\times \tau_{12}$ for an intrinsically brittle material (Adapted from [57]).

From the experimental results obtained for unidirectional fiber reinforced composite materials in [54], here categorized as intrinsically brittle materials according to Table 2, Equation (2a), Equation (2b) can substantially simplify the implementation for a given plane stress-state since no numerical search of the fracture plane is required. According to the experimental results in [54], it was verified that a plane stress case $\left(\sigma_{22},\tau_{12}\right)$ led to a fracture plane $\theta_{fp}=0{}^{\circ}$ when subject to any $\sigma_{22}\geq 0$. When subjected to $\sigma_{22}<0$, Puck and Schürmann [54] also found that there is a part of the fracture envelope where the fracture plane $\theta_{fp}=0{}^{\circ}$. According to them, the fracture is originated by $\tau_{12}$ whilst $\sigma_{22}$ impedes fracture. In the other part of the compression side, the fracture plane changes from $\theta_{fp}=0{}^{\circ}$ to $\theta_{fp}\neq 0{}^{\circ}$. Thus, although based on a simplified form of the 3D criterion, the derived 2D formulation requires a separate inspection of three modes on the stress space $\sigma_{22}\times \tau_{12}$ as can be seen in Figure 4. More details about this simplification, which includes surveying and distinguishing the fracture conditions between Mode A, B and C, can be found in [54,55]. Although the failure envelope is not symmetric to any vertical line, it has a symmetry with respect to the abscissa axis. Therefore, in Figure 4 the fracture curve is shown only for positive values of $\tau_{12}$.

Thus, taking into account the three separated modes of fracture in Figure 4, the fracture conditions in terms of the strength values and inclination parameters, subject to $\sigma_{22}$ and $\tau_{12}$ are written as [54,55]:

- Mode A, when $\sigma_{22}\geq 0$:
  (4) $$\sqrt{{\left(\frac{1}{Y_{T}}-\frac{p_{⊥\|}^{t}}{S_{12}}\right)}^{2}\sigma_{22}^{2}+{\left(\frac{\tau_{12}}{S_{12}}\right)}^{2}}+p_{⊥\|}^{t}\frac{\sigma_{22}}{S_{12}}=1,$$
- Mode B, when $\sigma_{22}<0$ and $0\leq \left|\frac{\sigma_{22}}{S_{12}}\right|\leq \frac{R_{⊥⊥}^{A}}{\left|\tau_{12C}\right|}$:
  (5) $$\sqrt{{\left(\frac{\tau_{12}}{S_{12}}\right)}^{2}+{\left(p_{⊥\|}^{c}\frac{\sigma_{22}}{S_{12}}\right)}^{2}}+p_{⊥\|}^{c}\frac{\sigma_{22}}{S_{12}}=1,$$
- Mode C, when $\sigma_{22}<0$ and $0\leq \left|\frac{S_{12}}{\sigma_{22}}\right|\leq \frac{\left|\tau_{12C}\right|}{R_{⊥⊥}^{A}}$:
  (6) $$\left[\frac{\tau_{12}^{2}}{4S_{12}^{2}{\left(1+p_{⊥⊥}^{c}\right)}^{2}}+\frac{\sigma_{22}^{2}}{Y_{C}^{2}}\right]\frac{Y_{C}}{-\sigma_{22}}=1,$$
  where
  (7) $$\tau_{12C}=\tau_{12}\sqrt{1+2p_{⊥⊥}^{c}}.$$

### 2.2. Applying Original Puck and Schürmann Inter-Fiber Fracture Criterion

**Figure 5:**
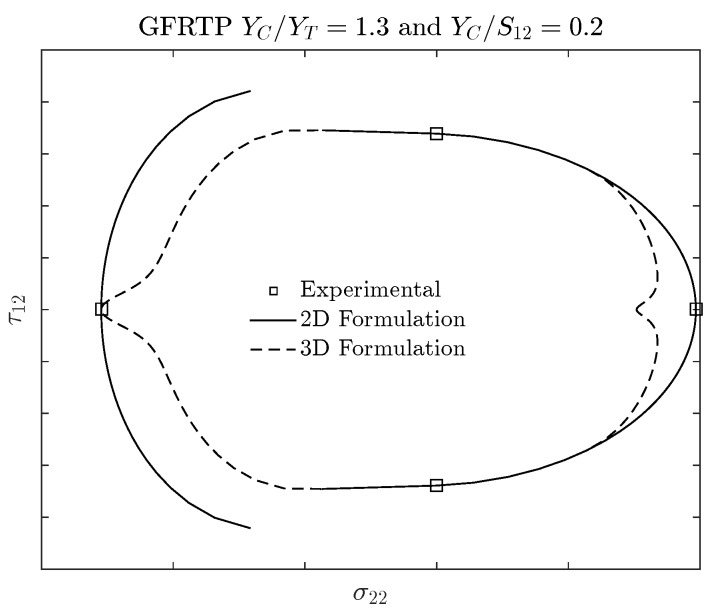
Failure envelopes computed for a 3D-printed glass-fiber composite material using both Puck and Schürmann 2D and 3D formulation.

In order to extend the application of original Puck and Schürmann Inter-Fiber Fracture Criterion to 3D-printed composite materials, a glass-fiber reinforced thermoplastic with ratios $Y_{C}/Y_{T}=1.3$ and $Y_{C}/S_{12}=0.2$ is used. According to the literature review, these ratios present common values found in the domain of fiber reinforced thermoplastic 3D-printed composite materials [5,15,35,40]. In Figure 5 the failure envelopes obtained for this material are presented. The inclination parameters $p_{⊥\|}^{c}$ and $p_{⊥\|}^{t}$ are the same as listed in Table 1 for GFRP/Epoxy composites. For the other two inclination parameters, it was adopted $p_{⊥⊥}^{c}=0.25$ and $p_{⊥⊥}^{t}=0.25$. Although a 2D stress-state was considered in order to plot these failure envelopes, both 2D and 3D formulations were used. It should be noted that the fracture planes were both assumed and computed according to the respective formulation.

It can be verified in Figure 5 that using the 2D Formulation, the transition point between Mode B and Mode C is not well defined. It means that the curves generated by both modes do not present a tangent nor coincident point. This is evidenced in Figure 5 by the discontinuity between the curves representing the 2D Formulation which are supposed to be continuous and smooth according to the Puck and Schürmann failure criterion. This can lead, for example, to a numerical problem when implementing the method into a FEM analysis, i.e., depending on the combined applied stress and the required precision, it may occur a pseudo-infinite loop—a very long loop which appears to be infinite—between the regions where the modes are not coincident. During the analyses the authors observed that lower values for the inclination parameters leads to a reduced discontinuous region between Modes B and C in failure envelopes computed using the 2D Formulation. However, even with values lower than the inferior limit remarked by Knops [57], the failure envelope still presented a discontinuous region between Mode B and Mode C.

In regards to the failure envelope computed using the 3D Formulation, it can be verified from Figure 5 that the curves seem to be continuous. However, the 3D Formulation was not able to capture the experimental tensile strength viewing that the highest stress exposure is being computed in a plane with a fracture angle $\theta_{fp}\neq 0{}^{\circ}$. Analogously to the 2D Formulation, lower values for the inclination parameters can reduce the distance of the failure envelope to the experimental tensile point but do not eliminate it. Moreover, the region under compressive loading of the failure envelope computed using 3D Formulation is particularly more conservative than the same region computed using 2D Formulation. Since 3D-printed materials are also known for their high potential to be optimized, this particularly more conservative failure envelope substantially reduces the feasible design region.

In summary, it can be verified that the Original Puck and Schürmann Inter-Fibre Fracture criterion should be adapted to predict the failure envelopes of 3D-printed materials whose ratios $Y_{C}/Y_{T}$ and $Y_{C}/S_{12}$ are similar to those presented in the example above. In other words, it should be adapted in order to provide continuous and smooth failure envelopes which also capture the experimental points and respect the limits for the inclination parameters that do not impose unrealistic compression fracture angles. In the next section an expansion on the Original Puck and Schürmann Inter-Fibre Fracture criterion is presented which overcome these points.

## 3. Expanded Puck and Schürmann Inter-Fiber Fracture Criterion for 3D-Printed Composite Materials

**Figure 6:**
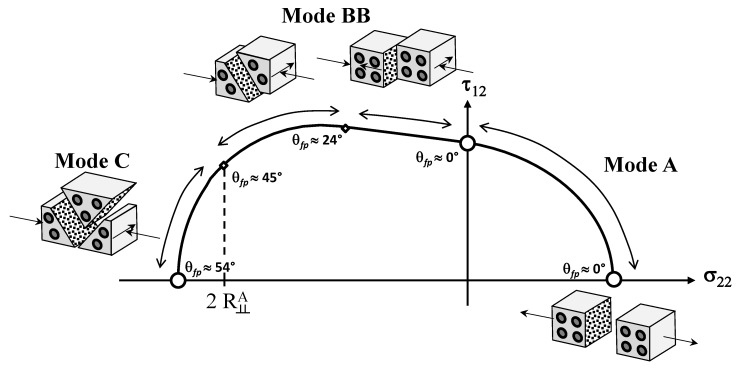
Proposed fracture curve on the stress space $\sigma_{22}\times \tau_{12}$.

In order to circumvent the points highlighted in the previous section, e.g., discontinuous and non-smooth curves, non-capture of the experimental points and reduced feasible design region, an expansion on the original Puck and Schürmann Inter-Fiber Fracture Criterion is then required for fiber reinforced thermoplastic 3D-printed composite materials. This section presents this expansion, hereinafter referred to as Expanded Puck and Schürmann (ExPan), as well as its assumptions. According to Puck and Schürmann theory [54,55], the fracture angles $\theta_{fp}\approx 45{}^{\circ}$ appear for intrinsically brittle materials under uniaxial transverse compressive load. Thus, Puck and Schürmann defined a point of transition at which the fracture condition passes from the Mode B ($\theta_{fp}=0{}^{\circ}$) to Mode C ($\theta_{fp}\neq 0{}^{\circ}$). However, the authors observed that, depending on the ratio $Y_{C}/S_{12}$, a pressure induced shear fracture condition of Mode B with $\theta_{fp}\neq 0{}^{\circ}$ can also appear. In other words, it can be said that for the region on the stress space $\sigma_{22}\times \tau_{12}$ which corresponds to this mode, henceforth named as Mode BB (see Figure 6), the fracture condition must be implemented and checked for every angle within the interval [−90°,90°]. It is worth mentioning that the real interval is, in fact, restricted to a subset of this wide interval. However, the authors kept this wide interval in order to be coherent with the characteristics of a general envelope methodology. Thus, according to the proposed method, Equation (5) should not be used in this region and Equation (2b) should be considered.

Analogously to the original Puck and Schürmann fracture curves, those provided by the Expanded Puck and Schürmann also have symmetry only with respect to the abscissa axis. Therefore, in Figure 6 the fracture curve is shown only for positive values of $\tau_{12}$. It can be observed from Figure 6 the relations between the failure conditions and the fracture angles. In Mode A, the fracture angle is assumed to be constant, although the failure condition switches from a pure tension to a pure shear failure. In Mode BB, it is verified a transition between different fracture angles while the fracture condition remains the same, i.e., even though a pure shear fracture is converted into a pressure induced shear fracture, it still remains a shear failure. Lastly, from Mode BB to Mode C a different fracture condition can be observed, where transverse compressive stress influences the fracture behavior which also includes changes in the fracture angle.

During the formulation for the Expanded Puck and Schürmann, the authors verified that this new transition point, i.e., the fracture condition passing from Mode BB to Mode C, corresponds to $\sigma_{22}=2R_{⊥⊥}^{A}$ as presented in Figure 6. The reason which lead the authors to this different approach is that the Mode B presented by Puck and Schürmann is not sensitive to the parameter $p_{⊥⊥}^{c}$. Thus, depending on the ratio $Y_{C}/S_{12}$, an anticipation of this transition to a fracture angle $\theta_{fp}\neq 0$ may appear but also maintain the fracture condition. It is not altogether irrelevant to note that this ratio $Y_{C}/S_{12}$ also may lead to some contradictions if, after this transition point, Equation (2b) continues to be used. This can be explained by the sensitivity of the region in the Mode C to the inclination parameter $p_{⊥⊥}^{c}$. In addition, it can also be shown that assuming $p_{⊥⊥}^{c}=p_{⊥⊥}^{t}$, as adopted in [56,71], will provide only expected responses.

The Expanded Puck and Schürmann method presented in this paper, assumes that a 2D stress-state is acting over the 3D-printed composite material. Nevertheless, it applies a set of equations derived for 3D stress-state in addition to a set of equations simplified for a 2D stress-state. In the context of 3D-printed composite materials it is very plausible to adopt this approach viewing that the deposited layers are very thin and behave as an orthotropic lamina [5,32,33,34,35,36,37,38,39,40]. In any case, the method is able to provide the failure envelopes for other laminated composite materials as can be seen in the next section.

### 3.1. Summary of the Failure Conditions and Respective Equations

In an attempt of facilitating the implementation of the Expanded Puck and Schürmann, the failure conditions are listed below as well as their proper equations and intervals of validity. The coefficients and parameters are the same as previously listed.

- Mode A, when $\sigma_{22}\geq 0$:
  (8) $$\sqrt{{\left(\frac{1}{Y_{T}}-\frac{p_{⊥\|}^{t}}{S_{12}}\right)}^{2}\sigma_{22}^{2}+{\left(\frac{\tau_{12}}{S_{12}}\right)}^{2}}+p_{⊥\|}^{t}\frac{\sigma_{22}}{S_{12}}=1,$$
- Mode BB, when $\sigma_{22}<0$ and $\left|\sigma_{22}\right|<2R_{⊥⊥}^{A}$. In this case, the fracture angle must be computed for the highest stress exposure:
  (9) $$f_{E}\left(\theta \right)=\sqrt{{\left[\frac{p_{⊥\psi }^{c}}{R_{⊥\psi }^{A}}\sigma_{n}\left(\theta \right)\right]}^{2}+{\left[\frac{\tau_{nt}\left(\theta \right)}{R_{⊥⊥}^{A}}\right]}^{2}+{\left[\frac{\tau_{n1}\left(\theta \right)}{S_{12}}\right]}^{2}}+\frac{p_{⊥\psi }^{c}}{R_{⊥\psi }^{A}}\sigma_{n}\left(\theta \right),$$
- Mode C, when $\sigma_{22}<0$ and $\left|\sigma_{22}\right|>2R_{⊥⊥}^{A}$:
  (10) $$\left[\frac{\tau_{12}^{2}}{4S_{12}^{2}{\left(1+p_{⊥⊥}^{c}\right)}^{2}}+\frac{\sigma_{22}^{2}}{Y_{C}^{2}}\right]\frac{Y_{C}}{-\sigma_{22}}=1.$$

### 3.2. Effects of $p_{⊥⊥}^{c}$ on the Expanded Puck and Schürmann

**Figure 7:**
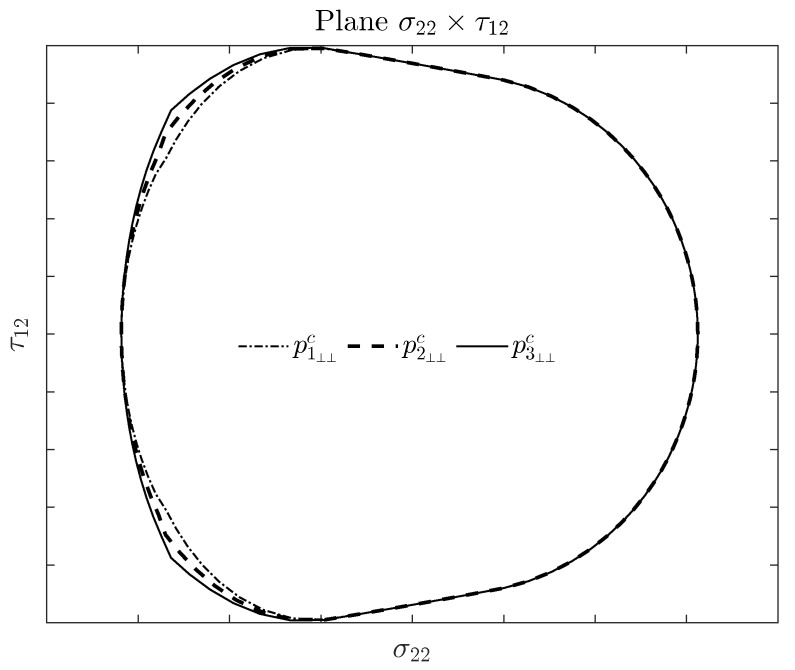
Effect of the inclination parameter $p_{⊥⊥}^{c}$ on the stress space $\sigma_{22}\times \tau_{12}$ failure envelope. This envelope was generated by the Expanded Puck and Schürmann criterion for a material with ratios $Y_{C}/Y_{T}=1.97$ and $Y_{C}/S_{12}=0.95$. In this figure $p_{3_{⊥⊥}}^{c}=0.3$, $p_{2_{⊥⊥}}^{c}=0.25$ and $p_{1_{⊥⊥}}^{c}=0.2$.

As demonstrated in this paper, the Expanded Puck and Schürmann can be accurately applied to materials with different ratios of $Y_{C}/Y_{T}$ and $Y_{C}/S_{12}$. It should be said that this certainly includes the intrinsically brittle materials. In this context, the choice of the value of the inclination parameter $p_{⊥⊥}^{c}$ may be used as driving point that makes the envelope more or less conservative. In other words, it can be interpreted as a parameter that makes the failure envelope fit the experimental points better, since they were measured from bi-axial tests. In Figure 7 it is illustrated how this parameter affects the failure envelope according to the Expanded Puck and Schürmann. It is worth remarking that the lower limit of $p_{⊥⊥}^{c}=0.2$ [54,55] should be respected [57].

## 4. Failure Envelopes for 3D-Printed Materials

In this section the failure envelopes are presented and have been computed for different thermoplastics 3D-printed materials (unreinforced and fiber reinforced), and also different ratios $Y_{C}/Y_{T}$ and $Y_{C}/S_{12}$, using the Expanded Puck and Schürmann. The 3D-printed materials are then classified according to their ratios $Y_{C}/Y_{T}$ as presented by Gu and Chen in [71]. The obtained results are compared to those provided by original Puck and Schürmann 2D formulation [54,55] and also compared to those provided by Gu and Chen [71].

### 4.1. 3D-Printed Continuous Carbon Fiber Reinforced Thermoplastic

The transverse and in-plane shear strengths of the 3D-printed continuous carbon fiber reinforced composite material [35,40] are presented in Table 3. This material has a ratio $Y_{C}/Y_{T}\approx 2$, which means that it can be classified as a semi-brittle material (see Table 2).

**Figure 8:**
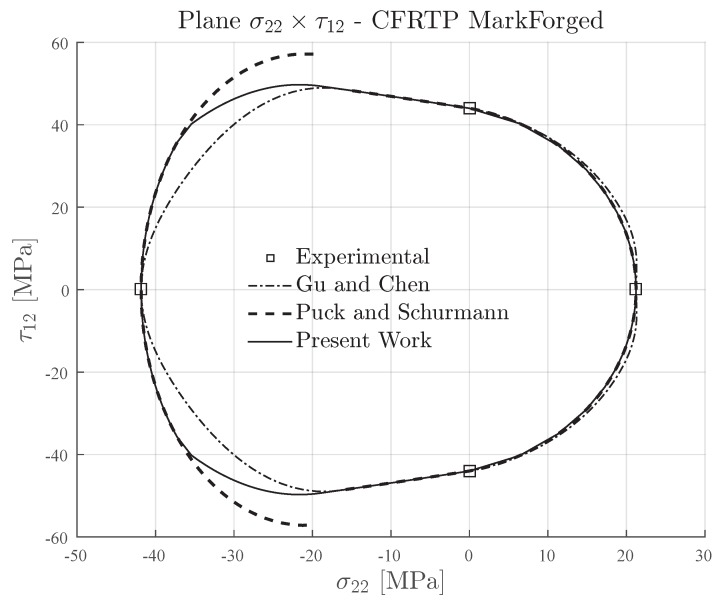
Failure envelopes on the stress space $\sigma_{22}\times \tau_{12}$ for 3D-printed continuous carbon fiber reinforced thermoplastic lamina. Inclination parameter $p_{⊥⊥}^{c}=0.3$ for Puck and Schürmann and Expanded Puck and Schürmann.

**Figure 9:**
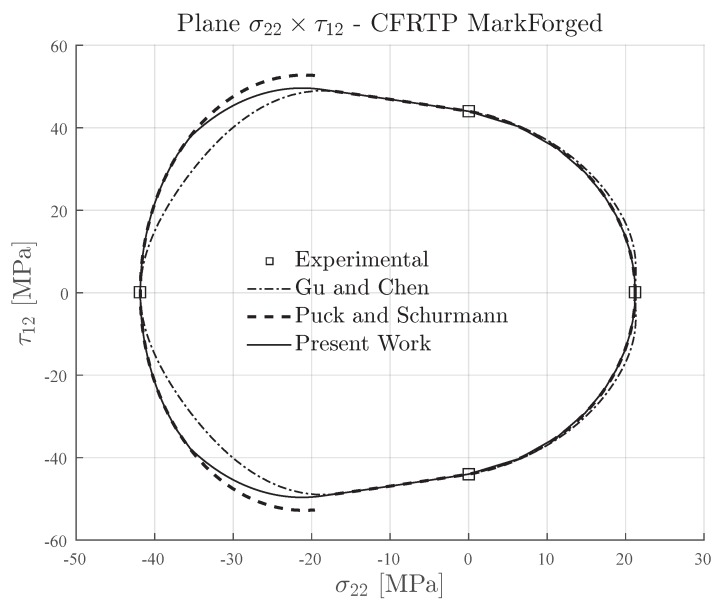
Failure envelopes on the stress space $\sigma_{22}\times \tau_{12}$ for 3D-printed continuous carbon fiber reinforced thermoplastic lamina. Inclination parameter $p_{⊥⊥}^{c}=0.2$ for Puck and Schürmann and Expanded Puck and Schürmann.

Figure 8 and Figure 9 display the computed failures envelopes of 3D-printed continuous carbon fiber reinforced composite material for the inclination parameters $p_{⊥⊥}^{c}=0.3$ and $p_{⊥⊥}^{c}=0.2$ respectively. The determination of these values were based on the recommendations for carbon fiber reinforced composite materials and also the lower limit mentioned by Knops [57]. For Gu and Chen failure envelopes, the inclination parameter $p_{⊥⊥}^{c}$ was computed according to their method proposed in [71].

It can be seen in Figure 8 and Figure 9 that the Expanded Puck and Schürmann provided a less conservative failure envelope than that provided by Gu and Chen. Taking into account experimental testing data for traditional composite materials [67], the stress-state on this region of the stress space $\sigma_{22}\times \tau_{12}$ is expected to be closer to the one predicted by the Expanded Puck and Schürmann than the one predicted by Gu and Chen. Compared to the Puck and Schürmann failure envelopes, the Expanded Puck and Schürmann appears to be in good agreement and has the advantage of creating a smooth and continuous transition between the fracture conditions under combined compressive transverse and in-plane shear loading.

### 4.2. 3D-Printed Continuous Glass-Fiber Reinforced Thermoplastic

The transverse and in-plane shear strengths of 3D-printed continuous glass-fiber reinforced composite material [35] are presented in Table 4. This material has a ratio $Y_{C}/Y_{T}\approx 1.3$, which means that it can also be classified as a semi-brittle material (see Table 2) although its ratio is almost in the lower limit for this classification. An important point about this material is concerned to the ratio between the compressive strength and in-plane shear strength which is $Y_{C}/S_{12}\approx 0.2$. As previously mentioned, and also demonstrated herein, this ratio can notably affect the prediction of the failure envelopes according to the Puck and Schürmann based methods available in the literature.

**Figure 10:**
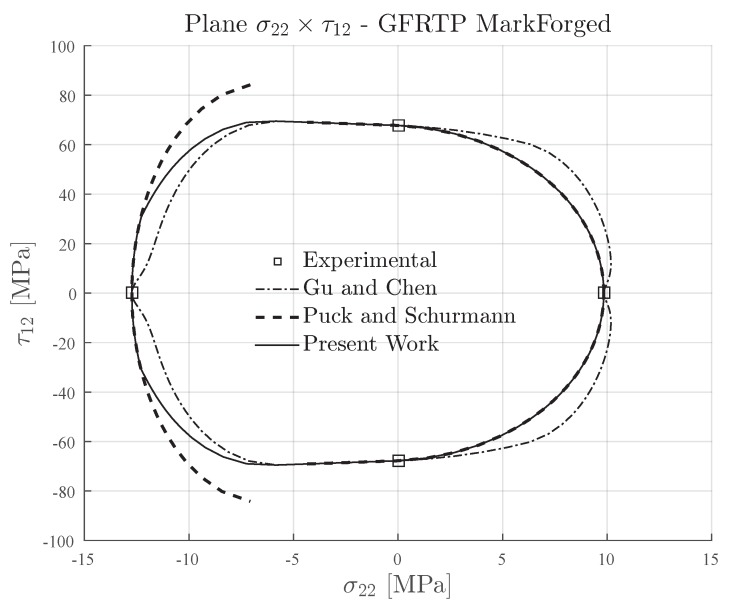
Failure envelopes on the stress space $\sigma_{22}\times \tau_{12}$ for 3D-printed continuous glass-fiber reinforced thermoplastic lamina. Inclination parameter $p_{⊥⊥}^{c}=0.25$ for Puck and Schürmann and Expanded Puck and Schürmann.

**Figure 11:**
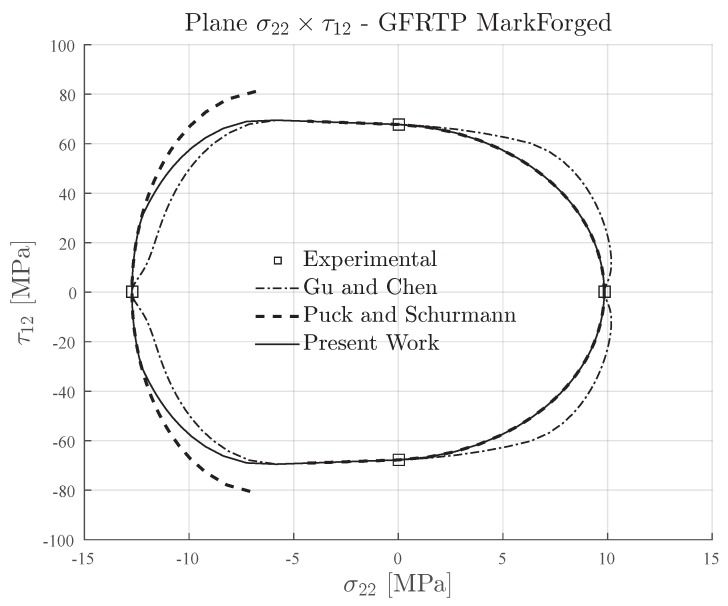
Failure envelopes on the stress space $\sigma_{22}\times \tau_{12}$ for 3D-printed continuous glass-fiber reinforced thermoplastic lamina. Inclination parameter $p_{⊥⊥}^{c}=0.2$ for Puck and Schürmann and Expanded Puck and Schürmann.

Figure 10 and Figure 11 present the computed failures envelopes of 3D-printed continuous glass-fiber reinforced composite material for the inclination parameters $p_{⊥⊥}^{c}=0.25$ and $p_{⊥⊥}^{c}=0.2$ respectively. The determination of these values were also based on the recommendations for glass-fiber reinforced composite materials and taking into account the lower limit mentioned by Knops [57]. For Gu and Chen failure envelopes, the inclination parameter $p_{⊥⊥}^{c}$ was computed according to the proposed in [71].

In Figure 10 and Figure 11 it can be verified that the Expanded Puck and Schürmann provided interesting failure envelopes for both values of the inclination parameter $p_{⊥⊥}^{c}$. Comparing the regions of the obtained failure envelope to those presented for traditional glass-fiber composite materials [67], it can be inferred that the proposed method is able to provide more suitable results even if the material is close to the limit to be considered a semi-brittle material. As already expected, the original Puck and Schürmann failure envelope presents some discontinuous regions mostly for a higher inclination parameter, as can be seen in Figure 10.

For the Gu and Chen failure envelope, it can be seen in Figure 10 and Figure 11 that the extension formulated in [71] leads to a more evident distance between its failure envelope and the Original Puck and Schürmann failure envelope. The authors believe that this difference is a affected by the small ratio $Y_{C}/S12$ which was not taken into account in their formulation. In this context, it should be highlighted that the non-expected values obtained for combined loading lies in the region close to the abscissa axis. Moreover, it is worth noting that the Gu and Chen failure envelopes are more conservative for glass-fiber composite materials similar to that shown for carbon fiber composite material.

### 4.3. Unreinforced 3D-Printed Material

In order to provide more data for further discussion, although not in the context of reinforced materials, the failure envelopes have been plotted for an unreinforced 3D-printed material, namely PLA. The transverse and in-plane shear strengths of unreinforced 3D-printed PLA [15,43] are presented in Table 5. Although it is not a reinforced material, it can be seen in the literature that the deposited layers also behave as orthotropic lamina. Moreover, this material has a ratio $Y_{C}/Y_{T}\approx 2.1$, which could easily classify it as a semi-brittle material (see Table 2). Additionally, it can be mentioned that the ratio $Y_{C}/S_{12}\approx 5.4$ is significantly higher which leads to a less conservative predicted region under transverse compressive loading.

Investigations about the failure of unreinforced 3D-printed PLA using the Puck and Schürmann approach were unavailable in literature. Therefore, there were no recommended values for the proper inclination parameter $p_{⊥⊥}^{c}$. In this context, the authors applied the inclination parameter according to the following equation [56,57]:

(11) $$p_{⊥⊥}^{c}=\frac{1}{2}\left(\sqrt{1+2p_{⊥\|}^{c}\frac{Y_{C}}{S_{12}}}-1\right)$$

**Figure 12:**
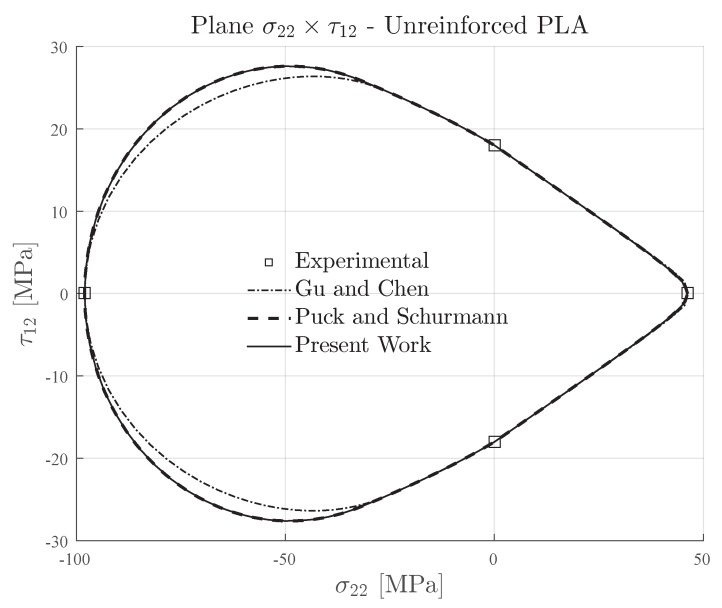
Failure envelopes on the stress space $\sigma_{22}\times \tau_{12}$ for unreinforced 3D-printed PLA. Inclination parameters $p_{⊥⊥}^{c}=0.53$ for Puck and Schürmann and Expanded Puck and Schürmann.

The obtained value for the inclination parameter $p_{⊥⊥}^{c}=0.53$ is greater than those proposed by Puck et al. [56] for traditional materials. It is completely out of the range originally established. However, since it was not previously investigated, the authors believe that it could be a good initial value. The failure envelopes are respectively presented in Figure 12. The Gu and Chen failure envelope was computed with the inclination $p_{⊥⊥}^{c}=0.31$ which was obtained according the theory proposed in [71]. It can be seen from Figure 12 that using the computed inclination parameter $p_{⊥⊥}^{c}=0.53$, the Expanded Puck and Schürmann provided good agreements with the failure envelopes of Puck and Schürmann and Gu and Chen. However, the Gu and Chen failure enveloped showed to be more conservative than the others.

## 5. Failure Envelopes for Traditional Composite Materials

In order to demonstrate that the Expanded Puck and Schürmann can still be applied for traditional composite materials, failure envelopes are presented for traditional semi-brittle, brittle and intrinsically brittle composite materials (see Table 2). Despite the fact that it was not in the scope of the present work to investigate the failure envelopes for traditional composite materials, it still remains a good demonstration that the Expanded Puck and Schürmann is general and can be used for a wide range of materials.

### 5.1. Traditional Semi-Brittle Materials

The transverse and in-plane shear strengths of IM7-8552 carbon fiber reinforced epoxy matrix [71] are presented in Table 6. This material has an in situ ratio $Y_{C}/Y_{T}\approx 1.23$, which means that it can be classified as a semi-brittle material (see Table 2). The ratio $Y_{C}/S_{12}\approx 1.5$ is not significantly high or low. It means that it is expected good agreement between the failure envelope computed for the Expanded Puck and Schürmann when compared to the Gu and Chen failure envelope.

**Figure 13:**
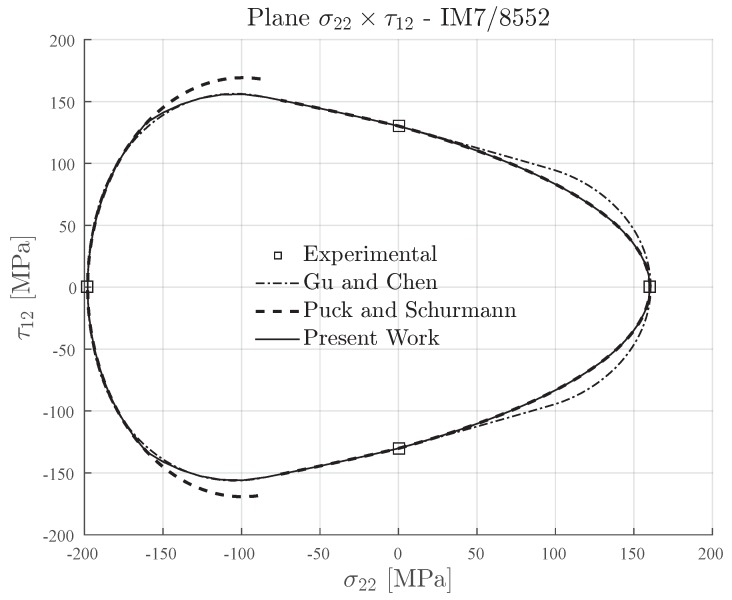
Failure envelopes on the stress space $\sigma_{22}\times \tau_{12}$ for IM7-8552. Inclination parameter $p_{⊥⊥}^{c}=0.3$ for Puck and Schürmann and Expanded Puck and Schürmann.

In Figure 13, the inclination parameter $p_{⊥⊥}^{c}=0.3$ was used for plotting the failure envelopes of Puck and Schürmann and also for the Expanded Puck and Schürmann. For the Gu and Chen failure envelope, the inclination parameter $p_{⊥⊥}^{c}$ was computed according to the formulation in [71]. It can be seen in Figure 13 that under transverse compressive loading, the Expanded Puck and Schürmann presented good agreement when compared to Gu and Chen. In the same region, the Puck and Schürmann failure envelope presented a discontinuity between Mode B and Mode C. Under transverse tensile loading, the Expanded Puck and Schürmann showed to be more conservative than the Gu and Chen.

### 5.2. Traditional Brittle Materials

The transverse and in-plane shear strengths of AS4-PEEK carbon fiber reinforced thermoplastic matrix [72] are presented in Table 7. This material has a ratio $Y_{C}/Y_{T}=2.5$, which means that it could be classified as a semi-brittle or brittle material since it is in the limit of the transition (see Table 2). The ratio $Y_{C}/S_{12}\approx 1.25$ is slightly low. This means that it can lead to some conservative regions under transverse compressive loading for the Gu and Chen failure envelope.

**Figure 14:**
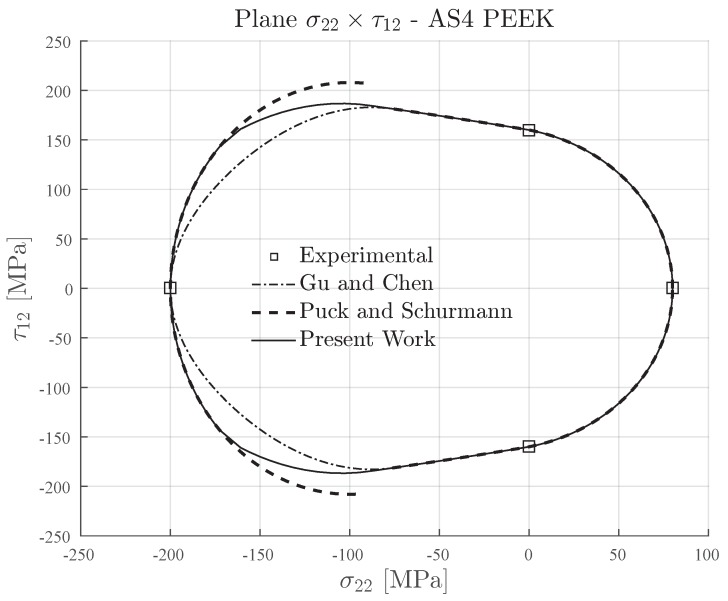
Failure envelopes on the stress space $\sigma_{22}\times \tau_{12}$ for AS4-PEEK. Inclination parameter $p_{⊥⊥}^{c}=0.3$ for Puck and Schürmann and Expanded Puck and Schürmann.

In Figure 14, the failure envelopes for AS4-PEEK material are shown. The inclination parameter $p_{⊥⊥}^{c}=0.3$ was used for plotting the failure envelopes of Puck and Schürmann and also for the Expanded Puck and Schürmann. For the Gu and Chen failure envelope, the inclination parameter $p_{⊥⊥}^{c}$ was computed according to the formulation in [71] for brittle materials. As already expected, it can be seen in Figure 14 that under transverse compressive loading, the Expanded Puck and Schürmann presented a less conservative failure envelope when compared to Gu and Chen. In the same region, the Puck and Schürmann failure envelope also presented a discontinuity between Mode B and Mode C. Under transverse tensile loading, the Expanded Puck and Schürmann presented a good agreement with the Gu and Chen prediction.

### 5.3. Traditional Intrinsically Brittle Materials

Lastly, it is shown in Table 8 the transverse and in-plane shear strengths of AS4-3506 carbon fiber reinforced epoxy matrix [67]. This material has a ratio $Y_{C}/Y_{T}\approx 4.5$, which means that it is an intrinsically brittle material (see Table 2). The ratio $Y_{C}/S_{12}\approx 3.75$ is considerable high when compared to the previous traditional composite materials, i.e., IM7-8552 and AS4-PEEK.

**Figure 15:**
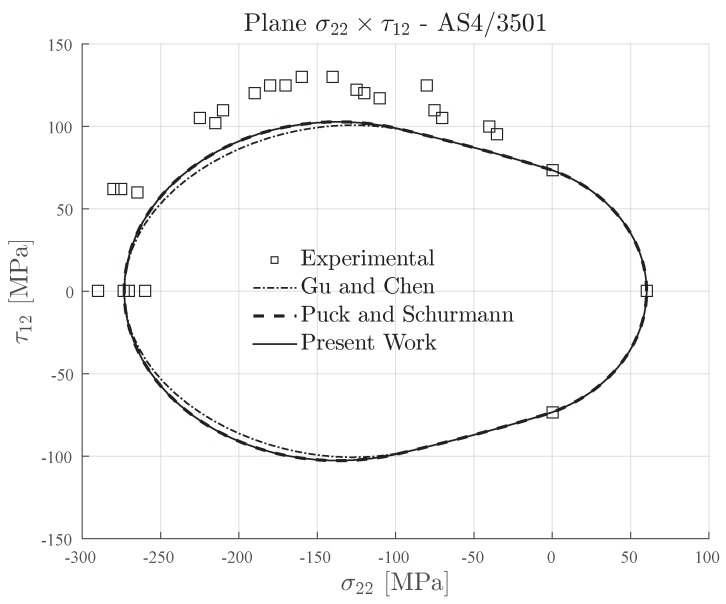
Failure envelopes on the stress space $\sigma_{22}\times \tau_{12}$ for AS4-3501 carbon fiber reinforced epoxy.

In Figure 15, it is shown the failure envelopes for AS4-PEEK material. For the Gu and Chen failure envelope, the inclination parameter $p_{⊥⊥}^{c}$ was computed according to the formulation in [71] for intrinsically brittle materials.

As already expected, all the plotted failure envelopes in Figure 15, i.e., the Expanded Puck and Schürmann, Puck and Schürmann and Gu and Chen, presented the same response in addition to a good agreement with the experimental data. This shows that the Expanded Puck and Schürmann can clearly be applied to conventional composite materials in addition to its ability of predicting the failure envelopes for 3D-printed reinforced materials.

## 6. Discussion

In the previous sections the failure envelopes were presented for both 3D-printed and traditional composite materials. It can be verified that the Expanded Puck and Schürmann provided smooth and continuous failure envelopes when compared to the Original Puck and Schürmann although a slight difference between the failure envelopes could be observed, mostly for the reinforced 3D-printed materials. It is worth remarking that a difference was also observed between the failure envelopes provided by Gu and Chen and the Original Puck and Schürmann. Viewing to provide more insight into the discussion, a geometrical analysis based on the distance between the failure envelopes was performed using the Original Puck and Schürmann as the reference. Since it was not found in the available literature reliable bi-axial testing data for reinforced and unreinforced 3D-printed materials, the authors believe that a geometrical comparison between the failure envelopes is an adequate first step to quantify their difference. Nowadays, there are several methods and tools to compute the geometrical difference between curves. Among them, it can be cited the polyline distance measure.

The distance between two polylines, here addressed as L1 and L2, is symmetrically defined as the average distance between a point of one polyline and the boundary of the other polyline [73,74]. Since that one polyline can have a different number of points compared to the other polyline, the distance d(p,s) between a point *p* and a line segment *s* needs to be evaluated. Thus, the distance d(p,s) between a point *p* with coordinates $\left(x_{0}^{p},y_{0}^{p}\right)$ and a line segment with end points $\left(x_{1}^{s},y_{1}^{s}\right)$ and $\left(x_{2}^{s},y_{2}^{s}\right)$ is defined as [73,74]:

(12) $$d\left(p,s\right)=\left\{\begin{array}{c} \min\{d_{1},d_{2}\}\;\mathrm{if}\;\lambda <0,\lambda >1,\\ \left|d^{⊥}\right|\;\mathrm{if}\;0\leq \lambda \leq 1, \end{array}\right.$$

where

(13a) $$\begin{array}{cc} \; & d_{1}=\sqrt{{\left(x_{0}^{p}-x_{1}^{s}\right)}^{2}+{\left(y_{0}^{p}-y_{1}^{s}\right)}^{2}}, \end{array}$$

(13b) $$\begin{array}{cc} \; & d_{2}=\sqrt{{\left(x_{0}^{p}-x_{2}^{s}\right)}^{2}+{\left(y_{0}^{p}-y_{2}^{s}\right)}^{2}}, \end{array}$$

(13c) $$\begin{array}{cc} \; & \lambda =\frac{\left(y_{2}^{s}-y_{1}^{s}\right)\left(y_{0}^{p}-y_{1}^{s}\right)+\left(x_{2}^{s}-x_{1}^{s}\right)\left(x_{0}^{p}-x_{1}^{s}\right)}{{\left(x_{2}^{s}-x_{1}^{s}\right)}^{2}+{\left(y_{2}^{s}-y_{1}^{s}\right)}^{2}}, \end{array}$$

(13d) $$\begin{array}{cc} \; & d^{⊥}=\frac{\left(y_{2}^{s}-y_{1}^{s}\right)\left(y_{0}^{p}-y_{1}^{s}\right)+\left(x_{2}^{s}-x_{1}^{s}\right)\left(x_{0}^{p}-x_{1}^{s}\right)}{\sqrt{{\left(x_{2}^{s}-x_{1}^{s}\right)}^{2}+{\left(y_{2}^{s}-y_{1}^{s}\right)}^{2}}}. \end{array}$$

The polyline distance $d_{L}\left(p,L_{2}\right)$ from point *p* to $L_{2}$ is defined by:

(14) $$d_{L}\left(p,L_{2}\right)=\min_{s\;\in \;L_{2}}d\left(p,s\right).$$

The distance $d_{pL}\left(L_{1},L_{2}\right)$ between the points of polyline $L_{1}$ and the boundary of polyline $L_{2}$ is defined as the sum of the distances from the points of the polyline $L_{1}$ to the closest segment/point of $L_{2}$:

(15) $$d_{pL}\left(L_{1},L_{2}\right)=\sum_{p\;\in \;\mathrm{points}\;L_{1}}d_{L}\left(p,L_{2}\right).$$

Reversing the computation from $L_{2}$ to $L_{1}$, the distance $d_{pL}\left(L_{2},L_{1}\right)$ is computed. Finally, the polyline distance between polylines $D_{s}\left(L_{2}:L_{1}\right)$ is defined by:

(16) $$D_{s}\left(L_{2}:L_{1}\right)=\frac{d_{pL}\left(L_{1},L_{2}\right)+d_{pL}\left(L_{2},L_{1}\right)}{N_{\mathrm{points}}\;\in L_{1}+N_{\mathrm{points}}\;\in L_{2}}.$$

From the point of view that the generated failure envelopes are composed by several line segments, Equation (16) was used to compute the polyline distance between them. The polyline distances $D_{s}$ between the failure envelopes computed for both 3D-printed materials and traditional composite materials are shown in Table 9 and Table 10 respectively. The distances are presented for the Expanded Puck and Schürmann in comparison to the Original Puck and Schürmann as well as for the Gu and Chen in comparison to the Original Puck and Schürmann. It can be verified that applying Equation (16) to compute the polyline distances between the failure envelopes, the distances $D_{s}$ in Table 9 and Table 10 have the same unit as the points computed to generate the failure envelope. In an attempt to provide a more clear understanding of these distances, the normalized distance $D_{s}/Y_{C}$ is also shown in Table 9 and Table 10 in addition to the distance $D_{s}$.

It can be observed from the normalized distances $D_{s}/Y_{C}$ in Table 9 and Table 10 that both the Expanded Puck and Schürmann method and the Gu and Chen method provided failure envelopes that are relatively close to the Original Puck and Schürmann, according to the polyline distance measure. However, the computed distances between the Expanded Puck and Schürmann and the Original Puck and Schürmann are smaller than the distances computed for the Gu and Chen in comparison to the Original Puck and Schürmann. This behavior is more prominent for the reinforced 3D-printed materials which are the object of study in this work. Additionally, it can be verified from Table 9 and Table 10 that the difference between the normalized distances $D_{s}/Y_{C}$ computed for the traditional composite materials in addition to 3D-printed PLA are smaller than the same distances computed for the reinforced 3D-printed materials.

As seen in Section 3.2, the choice of the inclination parameters, principally $p_{⊥⊥}^{t}$, may affect the results making the failure envelopes more or less conservative. This is evidenced with a closer analysis in the results obtained for 3D-printed reinforced composite materials (see Section 4). However, it can be verified from Table 9 that the Expanded Puck and Schürmann proposed in the present work, is not significantly affected by the inclination parameter if the recommended range of choice is respected, i.e., 0.2–0.3 for carbon fiber reinforced materials and 0.2–0.25 for glass fiber reinforced materials. For instance, taking into account the less conservative curves as references, i.e., those computed for smaller values of $p_{⊥⊥}^{t}$, it can be seen in Table 9 that the difference between the polyline distances is less than 6.5% for 3D-printed carbon fiber reinforced filaments and approximately 16% for 3D-printed glass fiber reinforced filaments. These results show that a misguided selection of this parameter would not significantly impact the overall results and, as mentioned in Section 4, it could be used as a parameter to correlate the failure envelopes with the experimental results.

In order to analyze the correlation between the normalized polyline distances and the ratio $Y_{C}/S_{12}$, the Spearman’s Rank-Order Correlation [75] was used to analyze the data in Table 9 and Table 10. Additionally, the same analysis was performed to verify the association between the normalized distances and the ratio $Y_{C}/Y_{T}$. In contrast to the Pearson’s Correlation [76], which evaluates the linear correlation between the variables, the Spearman’s Rank-Order Correlation is able to provide the correlation between two ranked variables as long as the correlation is monotonic, i.e., it is able to evaluate high orders correlations. It is worth noting that these methods are mostly used to verify the correlations between complete random variables which is not the case in this analysis. However, it still works as a suitable methodology to corroborate the assumptions adopted in this work. The Spearman’s correlation coefficient ρ is comprised in the interval [−1,1]. Values of ρ=1 or ρ=−1 indicates that the variables are in perfect monotonic positive or negative correlation respectively, i.e., the increment on the relation between the ranked variables is either always positive or negative. However ρ=0 indicates that the variables do not have a correlation, i.e., the closer ρ is to zero, the weaker the correlation between the ranked variables. The Spearman’s correlation coefficient ρ for tied ranks is defined as [75]:

(17) $$\rho =\frac{\sum_{i=1}^{n}\left(x_{i}-\bar{x}\right)\left(y_{i}-\bar{y}\right)}{\sqrt{\sum_{i=1}^{n}{\left(x_{i}-\bar{x}\right)}^{2}}\sqrt{\sum_{i=1}^{n}{\left(y_{i}-\bar{y}\right)}^{2}}},$$

where *n* is the variable size, *i* is the paired score, $x_{i}$ and $y_{i}$ are the individual tied ranks, $\bar{x}$ is the mean of *x* and $\bar{y}$ is the mean of *y*.

The results for the Spearman’s Rank-Order Correlation computed between the normalized polyline distances $D_{s}/Y_{C}$ and the ratio $Y_{C}/S_{12}$, as well as the correlation between the normalized polyline distances $D_{s}/Y_{C}$ and the ratio $Y_{C}/Y_{T}$, are shown in Table 11 for both Expanded Puck and Schürmann and Gu and Chen methods. In Table 11, ρ is the Spearman’s correlation coefficient and *p* is the marginal significance within a statistical hypothesis test representing the probability of the occurrence of a given event.

It can be seen from Table 11 that Spearman’s correlation coefficient indicates a negative correlation between the variables in both associations with the ratios $Y_{C}/Y_{T}$ and $Y_{C}/S_{12}$. For the association of $D_{s}/Y_{C}$ with $Y_{C}/S_{12}$ the values of ρ are substantially high and the marginal significance *p* are lower than the significance level of 0.05. This indicates the rejection of the null hypothesis, i.e., there exists a monotonic negative correlation between $D_{s}/Y_{C}$ and $Y_{C}/S_{12}$ and the results are statistically significant (p<0.05). However, for the association of $D_{s}/Y_{C}$ with $Y_{C}/Y_{T}$ the correlation coefficient ρ is not high which indicates a weak correlation between the variables. Furthermore, the marginal significance *p* are higher than the significance level of 0.05. Thus the null hypothesis shall not be rejected indicating that the weak correlation between $D_{s}/Y_{C}$ and $Y_{C}/Y_{T}$ is also not monotonic.

## 7. Conclusions

In the present work an Expanded Puck and Schürmann Inter-Fiber Fracture criterion was described in order to predict the failure envelopes of fiber reinforced thermoplastic 3D-printed composite materials. According to the literature reviewed, detailed studies about the failure of 3D-printed materials, as well as the recommended failure criteria, have not been conducted. Thus, the present work contributes in order to fill this gap in the literature. Furthermore, the method presented here is an expansion of Puck and Shürmann Inter-Fiber Fracture Criterion and is widely applicable in the composites field providing the required confidence, since it is based on phenomenological responses. It should be noted that, since the Puck and Schürmann failure criterion depends on the mechanical strength properties, among other parameters, it is important to the accuracy of the results the use of proper experimental data, either performing the tests or collecting data from the available literature. Due to this, the present investigation worked on the mechanical properties of 3D-printed materials collected from experimental characterizations that were carried out according to international standards, e.g., ASTM (American Society for Testing and Materials) and ISO (International Organization for Standardization), contributing for the accuracy of the obtained results.

In regards to the theoretical foundation of Expanded Puck and Schürmann, it was based on the assumption that the ratio between the transverse compressive strength and the in-plane shear strength should be taken into account when fiber reinforced thermoplastic 3D-printed composite materials are being investigated. The resulting failure envelopes were compared to those provided by the Original Puck and Schürmann in addition to those provided by Gu and Chen. In addition to presenting the failure envelopes for fiber reinforced thermoplastic 3D-printed composite materials, the present work also computed the failure envelopes for unreinforced 3D-printed material, since the deposited layers also behave as orthotropic lamina. In the context of Additive Manufacturing, failure characterization is a important step in order to contribute to innovative applications.

When compared to the Gu and Chen, the herein proposed Expanded Puck and Schürmann presents some advantages, e.g., it is not dependent of $S_{23}$ which is estimated, it does not have extra equations that need to be numerically solved and it also uses the inclination parameters as recommended by Puck and Schürmann. Moreover, the geometrical analysis demonstrated that the difference between the failure envelopes provided by Expanded Puck and Schürmann and by the Original Puck and Schürmann is lower than the difference between the failure envelopes provided by Gu and Chen and by the Original Puck and Schürmann. The performed statistical analysis showed that there is a strong correlation between the ratio $Y_{C}/S_{12}$ and the differences between the failure envelopes which corroborates the assumption that this ratio should be taken into account in the fracture criterion. Additionally, it was also demonstrated that the Expanded Puck and Schürmann is capable of providing suitable failure envelopes for traditional semi-brittle, brittle and intrinsically brittle composite materials, i.e., it is suitable not only for 3D-printed composite materials but also for any fiber reinforced thermoplastic/thermoset composite material. Finally, this study also shows the need for reliable bi-axial testing data of materials outside the use of the original method for further investigation and validation.

## Figures and Tables

**Table 1:** Typical values of inclination parameters for fiber reinforced plastics recommended by Puck et al. [56].

Material	$p_{⊥\|}^{t}$	$p_{⊥\|}^{c}$	$p_{⊥⊥}^{t}$	$p_{⊥⊥}^{c}$
Glass-Fiber/Epoxy	0.3	0.25	0.20–0.25	0.20–0.25
Carbon-Fiber/Epoxy	0.35	0.3	0.25–0.30	0.25–0.30

**Table 2:** Material classification according to different $Y_{C}/Y_{T}$ ratios presented by Gu and Chen in [71].

$Y_{C}/Y_{T}$	Classification
1–2.5	Semi-Brittle
2.5–3.45	Brittle
>3.45	Intrinsically Brittle

**Table 3:** Experimental data for 3D-printed continuous carbon fiber reinforced thermoplastic lamina [35,40].

Mechanical Property	Fiber Orientation	Value [MPa]
Tensile—$Y_{T}$	[90°]	21
Compression—$Y_{C}$	[90°]	41.8
In-Plane Shear—$S_{12}$	[45°/−45°]	44

**Table 4:** Experimental data for 3D-printed continuous glass-fiber reinforced thermoplastic lamina [35].

Mechanical Property	Fiber Orientation	Value [MPa]
Tensile—$Y_{T}$	[90°]	9.8
Compression—$Y_{C}$	[90°]	12.7
In-Plane Shear—$S_{12}$	[45°/−45°]	67

**Table 5:** Experimental data for unreinforced 3D-printed PLA [15,43].

Mechanical Property	Deposition Angle	Value [MPa]
Tensile—$Y_{T}$	[90°]	46.2
Compression—$Y_{C}$	[90°]	98
In-Plane Shear—$S_{12}$	[45°/−45°]	18

**Table 6:** Experimental data for IM7-8552 (in situ properties [71]).

Mechanical Property	Fiber Orientation	Value [MPa]
Tensile—$Y_{T}$	[90°]	160.2
Compression—$Y_{C}$	[90°]	198
In-Plane Shear—$S_{12}$	[45°/−45°]	130.2

**Table 7:** Experimental data for AS4-PEEK [72].

Mechanical Property	Fiber Orientation	Value [MPa]
Tensile—$Y_{T}$	[90°]	80
Compression—$Y_{C}$	[90°]	200
In-Plane Shear—$S_{12}$	[45°/−45°]	160

**Table 8:** Experimental data for AS4-3501 [67].

Mechanical Property	Fiber Orientation	Value [MPa]
Tensile—$Y_{T}$	[90°]	60.2
Compression—$Y_{C}$	[90°]	273.3
In-Plane Shear—$S_{12}$	[45°/−45°]	73.4

**Table 9:** Polyline distances (from Original Puck and Schürmann) computed for 3D-printed materials.

Material		Present Work		Gu and Chen	
		$D_{s}$	$D_{s}/Y_{C}$	$D_{s}$	$D_{s}/Y_{C}$
CFRTP	$p_{⊥⊥}^{c}=0.2$	0.408	0.97%	1.153	2.75%
	$p_{⊥⊥}^{c}=0.3$	0.435	1.04%	1.325	3.15%
GFRTP	$p_{⊥⊥}^{c}=0.2$	0.136	1.07%	0.367	2.88%
	$p_{⊥⊥}^{c}=0.25$	0.163	1.28%	0.389	3.05%
PLA		0.248	0.26%	0.317	0.33%

**Table 10:** Polyline distances (from Original Puck and Schürmann) computed for traditional composites.

Material	Present Work		Gu and Chen	
	$D_{s}$	$D_{s}/Y_{C}$	$D_{s}$	$D_{s}/Y_{C}$
IM7-8552	0.936	0.47%	1.242	0.63%
AS4-PEEK	1.039	0.52%	1.221	0.61%
AS4-3501	0.623	0.23%	0.765	0.28%

**Table 11:** Spearman’s Rank-Order Correlation computed between the normalized polyline distances $D_{s}/Y_{C}$ and the ratios $Y_{C}/S_{12}$ and $Y_{C}/Y_{T}$.

Normalized Distances	$Y_{C}/S_{12}$	$Y_{C}/Y_{T}$
Present Work—$D_{s}/Y_{C}$	ρ=−0.9639,p=0.0008	ρ=−0.5784,p=0.1419
Gu and Chen—$D_{s}/Y_{C}$	ρ=−0.8434,p=0.0127	ρ=−0.6266,p=0.1058
